# Nanoenergy and Nanosystems Advance the Development of Healthcare

**DOI:** 10.1002/advs.202507393

**Published:** 2025-05-11

**Authors:** Zhou Li, Heyi Wei

**Affiliations:** ^1^ Beijing Institute of Nanoenergy and Nanosystems Chinese Academy of Sciences Beijing 101400 China; ^2^ School of Nanoscience and Engineering University of Chinese Academy of Sciences Beijing 101400 China; ^3^ Tsinghua Changgung Hospital School of Clinical Medicine, School of Biomedical Engineering Tsinghua University Beijing 100084 China

The rapid development of nanoenergy and nanosystems has driven technological progress in the healthcare field. This special issue includes a total of 21 papers, among which 12 are review papers and 9 are research papers, demonstrating the transformation from material innovation to device breakthroughs and then to clinical applications.

Research on material innovation focuses on the development of electroactive materials (such as piezoelectric materials and triboelectric nanogenerators) and biodegradable materials. 2D piezoelectric materials improve energy harvesting efficiency. The symbiotic absorbable device prevents the device from remaining in the body and also avoids the need for secondary surgical removal. The polyvinyl alcohol aerogel reinforced with β‐lactoglobulin fibers significantly improved the performance of degradable implantable triboelectric nanogenerators. Furthermore, the development of mechanochemistry, by regulating the chemical behavior of substances through mechanical forces, has achieved control over reactions in solid‐state systems, thereby providing theoretical support for the development of new functional materials.

The development of new active materials has significantly improved the performance of functional medical devices. Microneedle technology enables painless transdermal drug delivery through dimensional and precise structural design. In addition, by reconstructing the microneedle electrode array system, subcutaneous multi‐parameter signal detection can be accomplished simultaneously. Through targeted modification of exosomes, such as CD16 antibody modification, environmentally responsive drug release is achieved, thereby enabling drug release through special barriers such as the retina and inner ear. In addition, injectable conductive hydrogels can intelligently release drugs based on ischemic environments, effectively improving myocardial repair.

Functional medical devices, on the one hand, promote tissue regeneration through electrical stimulation therapy, and on the other hand, achieve real‐time monitoring of physiological indicators by incorporating self‐powered sensors. Self‐powered sweat sensors, through environmental energy harvesting technology, and self‐powered pacemakers convert the mechanical energy of the heartbeat into electrical energy to drive the pacing electrodes, avoiding the implant surgery caused by the lifespan of traditional batteries. In the field of electrical stimulation therapy, precise epidural electrical stimulation (EES) was achieved through an implantable self‐powered system, effectively treating patients with spinal cord injury.

Although breakthroughs have been made in nanobiomedicine technology, issues such as the stability and output power of nanogenerators, the concern of biosafety, and the cost of nanomaterials have hindered their clinical transformation. Nowadays, with the popularization of artificial intelligence, this field urgently needs talents from multiple fields, such as artificial intelligence and computer science to join. For example, the Vision Transformer model extracts the features of the pulse signal and reduces the interference of motion artifacts. The deep integration of artificial intelligence has solved the problems of insufficient output power and signal interference of nanogenerators, which will be the key to breaking through the bottleneck.


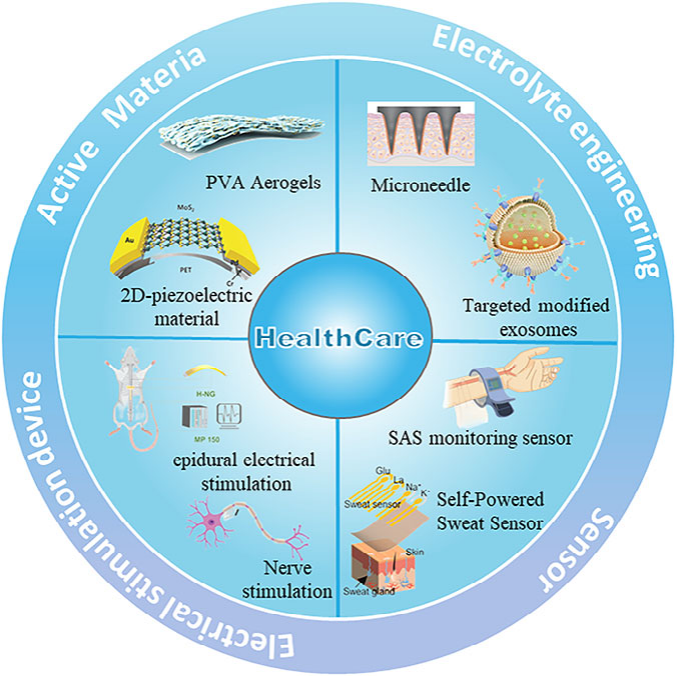


## Conflict of Interest

The authors declare no conflict of interest.

